# Integrated proteomic and metabolomic profiling identifies distinct molecular signatures and metabolic pathways associated with obesity and potential targets for anti-obesity therapies

**DOI:** 10.3389/fendo.2025.1625501

**Published:** 2025-07-31

**Authors:** Yi Li, Huawu Yang, Xinpeng Zhang, Xingyu He, Anke Liuli, Rui Li, Xingyu Han, Yongmei Li, Pan Gao

**Affiliations:** ^1^ Department of Radiology, The Third People’s Hospital of Chengdu, Chengdu, Sichuan, China; ^2^ Department of Radiology, The First Affiliated Hospital of Chongqing Medical University, Chongqing, China; ^3^ The Center of Obesity and Metabolic Diseases, Department of General Surgery, The Third People’s Hospital of Chengdu, Chengdu, Sichuan, China; ^4^ General Surgery Day Ward, Department of General Surgery, The Third People’s Hospital of Chengdu, Chengdu, Sichuan, China; ^5^ College of Life Science and Engineering, Southwest Jiaotong University, Chengdu, Sichuan, China; ^6^ School of Basic Medical Sciences, Southwest Medical University, Luzhou, Sichuan, China; ^7^ Obesity and Metabolism Medicine-Engineering Integration Laboratory, Department of General Surgery, The Third People’s Hospital of Chengdu, Chengdu, Sichuan, China

**Keywords:** obesity, adipose tissue, signature, metabolomic, proteomic

## Abstract

**Background:**

Adipose tissue remodeling induced by bariatric surgery plays a pivotal role in promoting weight loss and metabolic improvement. However, the underlying molecular mechanisms, particularly protein-metabolite regulatory networks, remain poorly understood. This integrative proteomic and metabolomic study identifies key pathway alterations and molecular signatures associated with metabolic phenotypes, offering novel mechanistic insights into the therapeutic efficacy of bariatric surgery.

**Methods:**

Visceral adipose tissue samples were analyzed using label-free DIA quantitative proteomics and LC-MS/MS metabolomics. Proteomic and metabolomic data were processed with MaxQuant software and XCMS R package, respectively.

**Results:**

Proteomic and metabolomic analyses were performed on visceral adipose tissue from 10 obese patients undergoing sleeve gastrectomy and 10 controls. Proteomic profiling quantified identified 135 differentially expressed proteins (57 upregulated, 78 downregulated), with PHACTR2 and PLIN2 upregulated in obesity and ADAR down-regulated in obesity. Enrichment analyses indicated disruptions in lipid droplet formation, muscle processes, and protein autophosphorylation, with KRT1/MYH9 and NF1/ATR identified as hub proteins. Metabolomics revealed 191 differential metabolites (110 upregulated, 81 downregulated), with 4-Vinylcyclohexene positively correlated with BMI and asparagine-betaxanthin negatively correlated. KEGG analysis showed disturbances in purine/pyrimidine metabolism, AMPK signaling, and cortisol biosynthesis. Integrated protein-metabolite network analysis identified OSBPL10, CUL2, and PRTN3 as potential regulators of lipid metabolism and insulin resistance, offering insights into obesity-associated metabolic dysfunction.

**Conclusions:**

This study integrated proteomic and metabolomic data from visceral adipose tissue obtained through sleeve gastrectomy, identifying obesity-related functional pathways and molecular signatures linked to metabolic phenotypes, highlighting the value of multi-omics in understanding adipose tissue remodeling and postoperative metabolic improvement.

## Introduction

Obesity has emerged as a major global public health challenge, currently affecting more than 878 million adults worldwide and closely associated with the development of numerous chronic diseases (1), including type 2 diabetes, cancer, and cardiovascular disorders (2–4). Bariatric surgery, particularly laparoscopic sleeve gastrectomy (SG), is recognized as one of the most effective clinical interventions for severe obesity, capable of inducing substantial weight loss and improving metabolic health (5). Although the clinical benefits of SG (including weight reduction, improvements in glycemic and lipid profiles) have been widely reported (6–8), the molecular adaptation mechanisms within adipose tissue that mediate these long-term metabolic improvements remain poorly understood.

As a critical organ regulating metabolic homeostasis, adipose tissue plays a central role in the development of obesity and its associated metabolic dysfunctions (9). In obesity, adipose tissue function becomes impaired, characterized by insulin resistance and dysregulated lipid metabolism (10, 11). Weight loss induced by bariatric surgery partially restores adipose tissue function and enhances insulin sensitivity (12). While these phenomena have been observed, a comprehensive understanding of the proteomic and metabolomic changes in adipose tissue following SG, as well as their associations with long-term metabolic outcomes, remains limited. Most existing multi-omics studies have focused primarily on blood samples or subcutaneous adipose tissue, while relatively little attention has been paid to visceral adipose tissue (VAT), which is closely associated with insulin resistance and metabolic risk.

Recent advances in proteomics and metabolomics have begun to uncover molecular pathway alterations associated with obesity and weight loss (13, 14). For example, studies have reported changes in sphingolipid metabolism and inflammatory signaling pathways following bariatric surgery (15). However, these studies often lack longitudinal clinical data and rarely integrate proteomic and metabolomic profiles with detailed postoperative phenotypic outcomes. In this study, we performed an integrated proteomic and metabolomic analysis of visceral adipose tissue from 10 obese patients undergoing SG and matched 10 healthy controls. By combining molecular data with one-year clinical follow-up information, we aimed to systematically characterize adipose tissue-specific pathway alterations associated with weight loss and metabolic improvement, and to identify potential molecular signatures linked to long-term outcomes. The uniqueness of this study lies in its first demonstration of the associations between adipose tissue proteomic and metabolomic signatures and clinical outcomes such as body mass index (BMI) reduction and ​metabolic dysfunction-associated steatotic liver disease (MASLD) remission after SG. These findings provide novel insights into the mechanisms of adipose tissue remodeling in the context of obesity intervention. Moreover, they deepen our understanding of the molecular basis of bariatric surgery and offer a foundation for individualized postoperative prognosis assessment and the development of new therapeutic targets.

## Materials and methods

### Subjects and study design

This study enrolled 30 subjects from the Third People’s Hospital of Chengdu, including 20 obese patients (8 males and 12 females) who underwent SG as part of the Longitudinal Study of Bariatric Surgery in Western China (registration number: ChiCTR2300073353), and another 10 healthy control participants (5 males and 5 females). All subjects met the following inclusion criteria: 1) obesity group (BMI > 28 kg/m²), control group (BMI < 24 kg/m²); 2) age between 18 and 65 years. Besides, we systematically excluded obese subjects diagnosed with polycystic ovary syndrome (PCOS). Within three days prior to surgery, several clinical phenotypic information was systematically collected for each subject, including BMI, total cholesterol (TC), low-density lipoprotein (LDL), high-density lipoprotein (HDL), triglycerides (TG), fasting blood glucose (Glu), aspartate aminotransferase (AST), alanine aminotransferase (ALT), uric acid, urea nitrogen, creatinine, and other metabolic indices, as well as clinical information such as history of MASLD and diabetes.

For postoperative follow-up, the 20 SG patients were monitored longitudinally for 1 year, with regular assessments of body weight. Venous blood samples were drawn at 1-year post-surgery, and several metabolic-related clinical parameters were measured to evaluate the metabolic improvement resulting from the surgical intervention. For adipose tissue sample selection, we prioritized obese patients with complete 1-year follow-up data to ensure prognostic reliability. To maintain balanced group sizes for comparative analysis, we ultimately selected 10 SG patients from the original 20 obese participants for VAT collection, matched with VAT samples from 10 controls. All tissue specimens were collected, processed, and stored under standardized protocols to ensure sample integrity and consistency. This study was conducted in strict accordance with the ethical principles of the Declaration of Helsinki. The study protocol was reviewed and approved by the Ethics Committee of Chengdu Third People’s Hospital.

### Samples collection

Standardized biospecimen collection and processing protocols were followed. All subjects fasted for 12 hours and abstained from medication for 24 hours prior to blood sampling. In the morning, trained medical personnel collected 5 mL of venous blood from each subject, and the samples were immediately stored at 4 °C. During the sleeve gastrectomy procedure, the surgical team collected omental adipose tissue samples according to standard procedures. Excised tissues were immediately transferred on dry ice and, under biosafety conditions, divided into two portions: one for DIA proteomic analysis (including library preparation and sample analysis) and one for untargeted metabolomic analysis. Any remaining tissue was processed and stored at -80 °C to ensure availability for future supplementary assays and validation studies. All procedures strictly followed standardized operating protocols to ensure sample quality and data reliability.

### Proteome analysis

Peptide and protein identification was performed using MaxQuant (v2.6.7) against the UniProt homo sapiens reference proteome database (16, 17). Key parameters were defined in the configuration file mqpar.xml, with the following settings: DIA and MS2-based quantification modes; spectral library-based quantification; label-free quantification enabled; enzyme specificity set according to the digestion protocol; and “Match Between Runs” enabled to reduce missing values across samples. Intensity-based absolute quantification (iBAQ) was used for protein abundance normalization. The false discovery rate (FDR) was controlled at 1% at both the peptide and protein levels. Identifications based on single peptides and low-quality spectra were excluded. Following identification, raw protein intensities were normalized using median centering and batch correction. Differential expression analysis was initially performed using unadjusted P < 0.05 and |log>FC| ≥ 1 as cutoffs. Proteins meeting these criteria were considered high-confidence differentially expressed proteins (DEPs).

### Metabolome analysis

The raw mass spectrometry data were first converted into mzXML format using ProteoWizard MSConvert (18). Subsequent data processing, including peak detection, retention time correction, peak alignment, and peak area extraction, was performed using the XCMS package (19). Specifically, peak detection was conducted using the centWave algorithm with the following parameters: ppm = 15 and peak width = 5–10 seconds. Peak alignment was carried out with a bandwidth (bw) of 5 and a binSize of 0.025. Ion features were retained for downstream analysis if more than 50% of samples in at least one group exhibited nonzero intensity measurements (20). Metabolites identification was performed according to the standards proposed by the Metabolomics Standards Initiative. Metabolite annotation was conducted by integrating information from the Human Metabolome Database (HMDB) and the Kyoto Encyclopedia of Genes and Genomes (KEGG) (21, 22), based on accurate mass matching with a mass error threshold of less than 5 ppm. Significantly altered metabolites were identified based on the following criteria: P < 0.05, and variable importance in projection (VIP) than 1.

### Pathway analysis and protein–protein interaction network

Functional enrichment analysis was performed to investigate the biological significance of the DEPs and metabolites. Gene Ontology (GO) enrichment analysis, covering Biological Process (BP), Cellular Component (CC), and Molecular Function (MF) categories, was conducted using the clusterProfiler R package (23). KEGG pathway analysis was similarly performed to identify significantly enriched pathways, with a P < 0.05 considered statistically significant. For protein-protein interaction (PPI) network construction, DEPs were input into the STRING database (v11.5) with a minimum interaction score threshold of 0.4 (24). The resulting network was visualized using Cytoscape software (v3.9.1) (25). Key hub proteins were identified based on network topology parameters such as degree centrality and betweenness centrality.

### Statistical analysis

All statistical analyses were conducted using R software (v4.3.2) in this study. Continuous variables were expressed as mean ± sd or median with interquartile range (IQR) as appropriate. Paired-sample t-tests or Wilcoxon signed-rank tests were used for pre- and post-surgical comparisons within the same group. Categorical variables were analyzed using the chi-square test. Pearson correlation coefficients were calculated to assess associations between molecular features (proteins or metabolites) and clinical phenotypes. For multiple comparisons, p-values were adjusted using the Benjamini-Hochberg FDR method.

## Results

### Clinical characteristics of participants

This study analyzed comprehensive clinical data from a cohort of 30 participants, comprising 20 obese patients undergoing SG and 10 healthy controls. The clinical data encompassing key metabolic indicators such as BMI, TC, LDL, HDL, TG, ALT, AST, Glu, uric acid, creatinine, urea nitrogen, and documented histories of MASLD and diabetes (**Supplementary Table S1**). Longitudinal follow-up data were obtained over 12 months for all surgical patients, capturing metabolic changes (n = 12) and weight trajectories (n = 20) (**Table 1**). Comparative analysis revealed several interesting findings. No significant differences were observed in age between the obese and control groups, minimizing potential demographic bias. As expected, preoperative BMI in the obese group was significantly higher than in controls, while the BMI values declined markedly postoperatively, approaching those of healthy individuals, demonstrating the efficacy of SG in inducing substantial weight loss.

**Table 1:** Clinical characteristics of participants before and after bariatric surgery and healthy controls.

Phenotypic information	Pre-surgery (n=20)	Post-surgery (n=12)	Controls (n=10)
Age (years)	33.25 ± 6.67	32.16 ± 7.12	40.50 ± 12.42
BMI (kg/m2)	36.19 ± 4.18	25.63 ± 3.59*	23.69 ± 2.73*
TC (mmol/L)	4.77 ± 1.01	4.74 ± 0.75	4.87 ± 0.77
LDL (mmol/L)	3.01 ± 0.91	2.85 ± 0.56	2.84 ± 0.74
HDL (mmol/L)	1.17 ± 0.30	1.40 ± 0.29*	1.56 ± 0.42*
TG (mmol/L)	2.32 ± 1.99	0.83/0.25*	1.50 ± 0.48
Glu (mmol/L)	5.69 ± 1.64	4.38 ± 0.42*	5.12 ± 0.94
ALT (U/L)	40.93 ± 31.87	13.01 ± 5.27*	64.9 ± 127.74
AST (U/L)	28.45 ± 18.12	15.6 ± 2.72*	51.36 ± 92.79
Uric acid (μmol/L)	423.75 ± 102.43	333.90 ± 59.30*	350.20 ± 105.40
Creatinine (μmol/L)	56.32 ± 15.63	61.25 ± 11.12	67.76 ± 15.33
Urea nitrogen (mmol/L)	8.72 ± 13.20	4.69 ± 1.52	4.40 ± 0.90
History of MASLD	20/20	6/12	0/20
History of diabetes	4/20	2/12	0/20

Values are shown as mean [mean ± sd]. *P* value: significance levels based on Wilcoxon signed rank test. Significant (*P* < 0.05) are marked * (Pre-surgery or Post-surgery vs. Controls). BMI, body mass index; TC, total cholesterol; LDL, low-density lipoprotein cholesterol; HDL, high-density lipoprotein cholesterol; TG, triglycerides; Glu, glucose; ALT, alanine aminotransferase; AST, aspartate aminotransferase; MASLD, non-alcoholic fatty liver disease; Pre-surgery, obesity before bariatric surgery; Post-surgery, obesity after bariatric surgery.

Compared to controls, obese patients exhibited significantly reduced HDL levels preoperatively, a finding that improved significantly after surgery. TG levels were elevated in the obesity group, although the difference did not reach statistical significance, and a marked reduction in TG levels was observed one year after SG. Moreover, postoperative improvements were evident across several biochemical parameters, including significant reductions in Glu, ALT, AST, and uric acid, suggesting systemic metabolic amelioration following weight loss surgery. Notably, all 20 obese participants presented with MASLD prior to SG. One year postoperatively, only 6 of the 12 followed-up patients continued to exhibit signs of hepatic steatosis, indicating partial reversal of MASLD in a subset of patients. These results underscore the therapeutic impact of SG not only on weight but also on broader metabolic health, including liver function and lipid regulation.

### Comprehensive proteomic changes in visceral adipose tissue between obesity and controls

**Figure 1:**
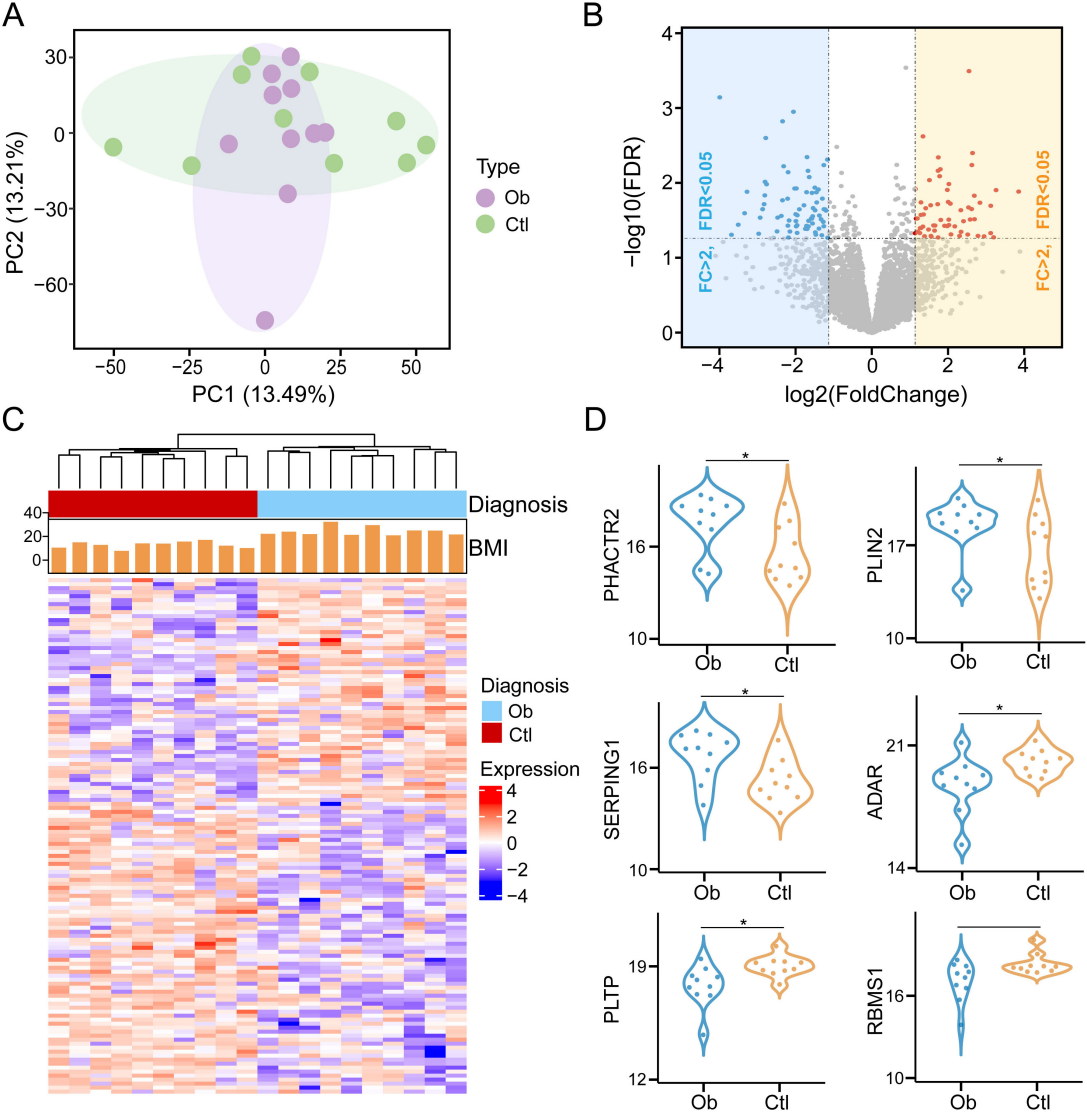
Identification of differentially expressed proteins in visceral adipose tissue between obesity post-bariatric surgery and healthy-weight control groups (Ob, Obesity; Ctr, Control). **(A)** Principal component analysis (PCA) of protein expression profiles across samples. **(B)** Volcano plot of DEPs with thresholds set at |log>FC| > 1 and P < 0.05. **(C)** Hierarchical clustering heatmap of DEPs. **(D)** Violin plots illustrating expression levels of representative DEPs between two groups. * means P<0.05.

To systematically investigate proteomic alterations in VAT associated with obesity, we performed label-free DIA quantitative proteomic profiling on VAT samples obtained from 10 obese patients undergoing bariatric surgery and 10 control individuals undergoing abdominal surgery for non-metabolic indications. A total of 4813 proteins were identified and quantified across all samples based on database search and stringent filtering criteria. Principal component analysis (PCA) based on the global proteomic profiles revealed a clear separation between the obesity and controls, with principal components 1 (PC1) and principal components 2 (PC2) explaining 13.49% and 13.2% of the total variance, respectively (**Figure 1A**). This suggests a robust divergence in protein expression patterns associated with the obese state. A total of 135 proteins were found to be differentially expressed between the two groups, meeting the criteria of statistical significance (P < 0.05) and a fold change threshold (|log<FC| > 1) (**Supplementary Table S2**). Of these, 57 proteins were upregulated and 78 were downregulated in the obese group relative to the controls (**Figure 1B**). Hierarchical clustering of these DEPs further demonstrated distinct expression signatures between obesity and controls (**Figure 1C**). Next, violin plots highlighted several representative proteins with significant expression differences. For example, PHACTR2, PLIN2, and SERPING1 were markedly upregulated in the obese group, whereas ADAR, PLTP and RBMS1 exhibited higher expression in controls (**Figure 1D**). These protein-level alterations may reflect key molecular mechanisms underlying VAT dysfunction in obesity and provide potential targets for further mechanistic studies.

### Protein functional pathways alterations associated with obesity

**Figure 2:**
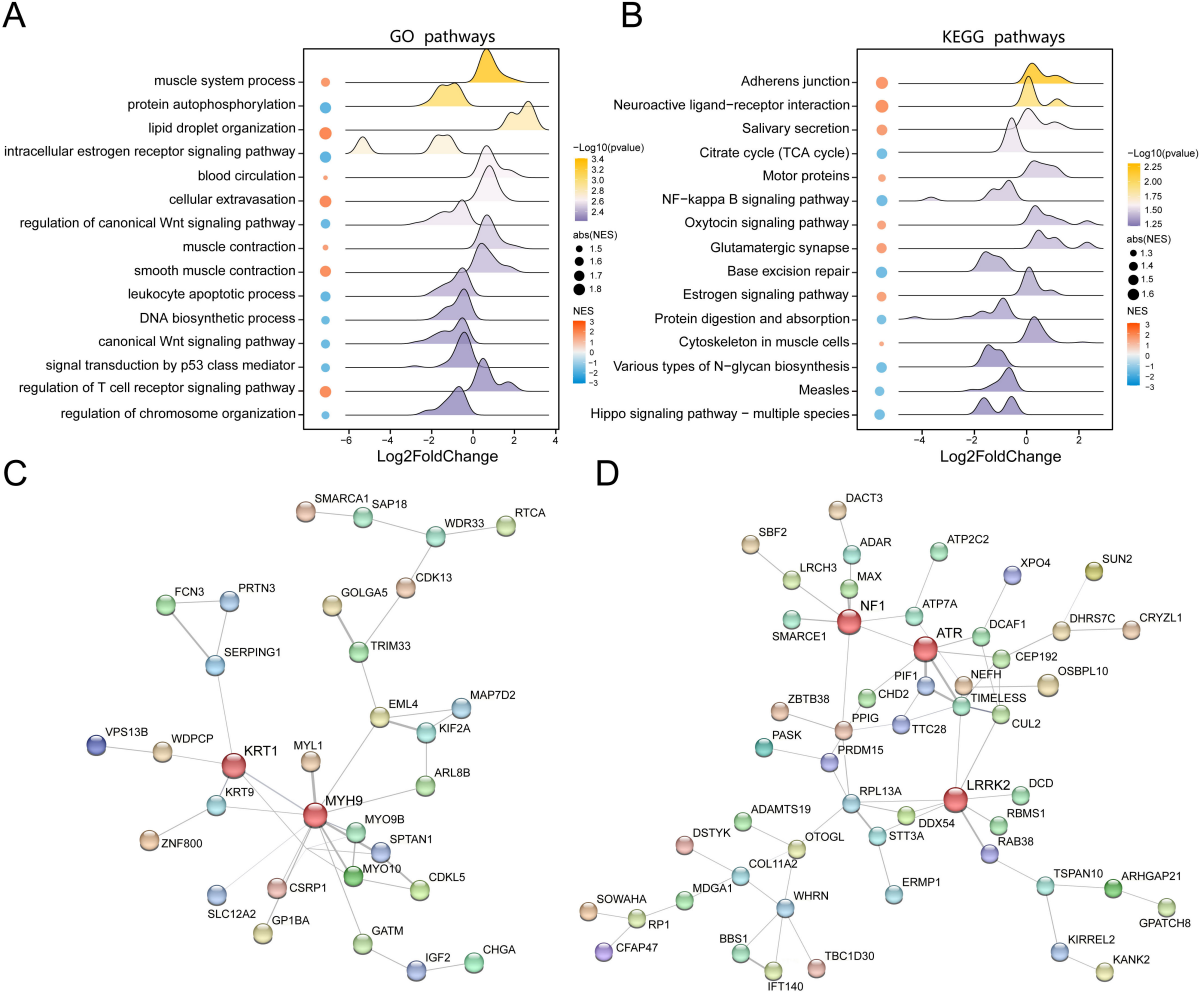
Functional enrichment analysis of DEPs between obesity and control groups. **(A)** Bubble chart of significantly enriched Gene Ontology terms based on DEPs. **(B)** Bubble chart of significantly enriched KEGG pathways. **(C)** Protein-protein interaction (PPI) network of upregulated proteins in obesity (nodes: proteins; edges: interactions; node color: expression fold change). **(D)** PPI network of downregulated proteins in obesity, highlighting key hub proteins.

To further elucidate the biological implications of proteomic alterations in VAT of obese individuals, we conducted GO and KEGG pathway enrichment analyses based on the set of DEPs. We showed the 15 representative GO biological processes that were significantly enriched (P < 0.05), indicating functional convergence of obesity-related proteins (**Figure 2A**, **Supplementary Table S3**). Of particular note was the lipid droplet organization pathway, indicating that lipid processing and remodeling in VAT were significantly affected in the obese state. Besides, our results also show that obesity-related DEPs are related to muscle system processes and protein autophosphorylation functions. In obesity, skeletal muscle undergoes tissue remodeling through ectopic lipid deposition and inflammatory response, leading to metabolic disorders, and is closely related to the development of insulin resistance and MASLD (26). In addition, the role of protein phosphatases is essential for a variety of physiological responses, and protein kinase-phosphoprotein interactions also play an important role in obesity (27). These findings are consistent with the central role of adipose tissue in systemic energy homeostasis and lipid storage. Similarly, we displayed the top 15 significantly KEGG pathways (**Figure 2B**, **Supplementary Table S4**), among which the top three pathways, adherens junction, endocrine system, and salivary secretion were upregulated in obesity compared to healthy controls. These pathways are functionally related to epithelial barrier dysfunction, appetite regulation, and salivary organ secretion in obesity (28–30), all of which are known to be altered in obesity-induced adipose tissue dysfunction.

To investigate the functional interplay among the DEPs, we constructed protein-protein interaction (PPI) networks for both the upregulated and downregulated protein subsets. The network of upregulated proteins demonstrated a dense interaction architecture with KRT1 and MYH9 emerging as key hub nodes, suggesting their potential core roles in the functional transformation of VAT in obesity (**Figure 2C**). In contrast, the PPI network of downregulated proteins highlighted NF1, ATR, and LRRK2 as major interaction hubs (**Figure 2D**), indicating proteins that play a key role in the lean state. These results collectively underscore the complexity and specificity of proteomic remodeling in VAT during obesity and reveal functional clusters that may contribute to adipose tissue pathology and systemic metabolic dysregulation.

### Untargeted metabolomic profiling of visceral adipose tissue with obesity

**Figure 3:**
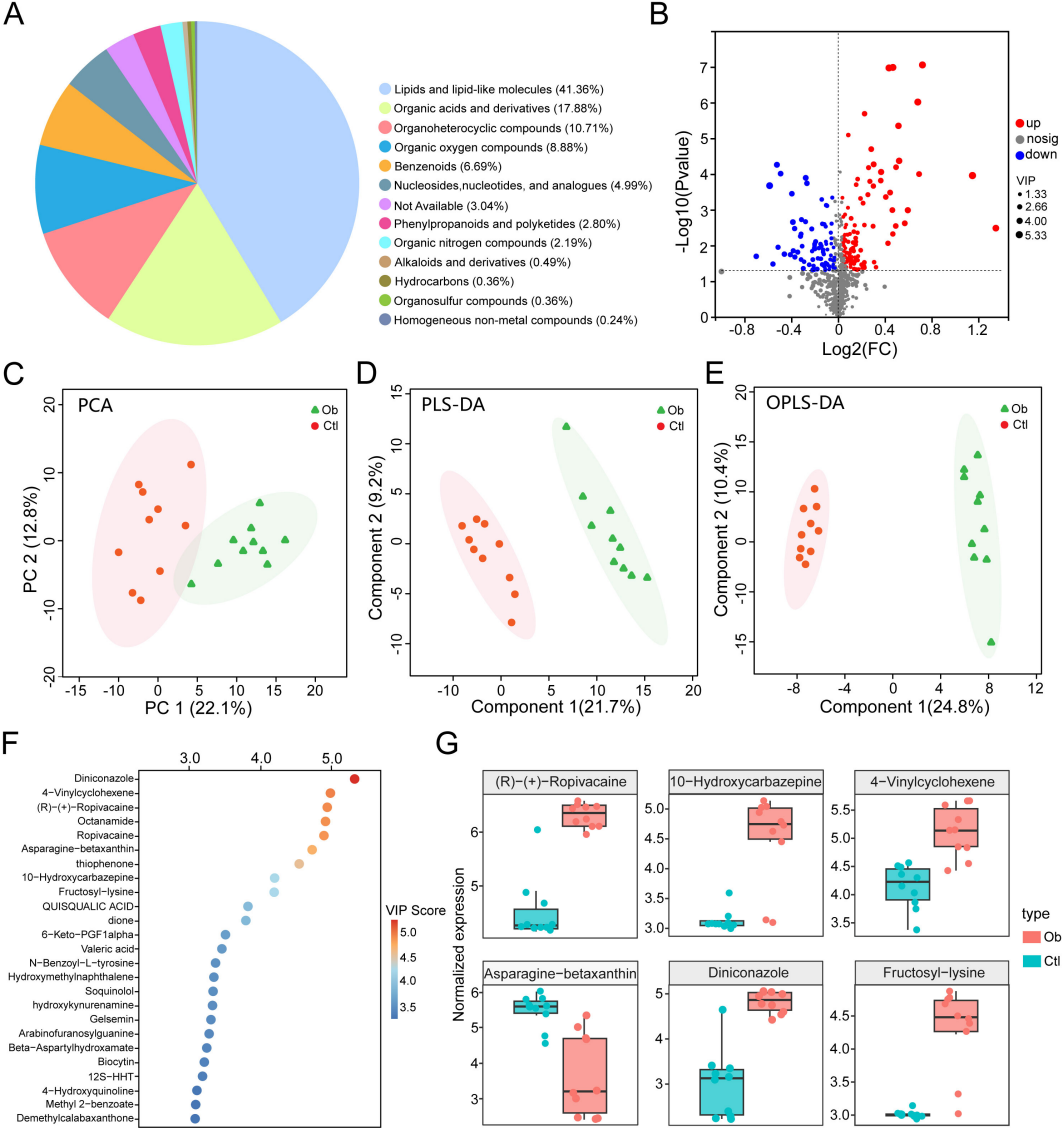
Liquid chromatography-mass spectrometry-based metabolomics analysis of obesity and controls groups. **(A)** Pie chart of chemical taxonomy super classes annotated using the Human Metabolome Database. **(B)** Volcano plot of differential metabolites (VIP > 1.0, P < 0.05). **(C)** PCA score plot of metabolite expression profiles. **(D)** Partial least squares-discriminant analysis (PLS-DA) score plot. **(E)** Orthogonal PLS-DA (OPLS-DA) score plot. **(F)** Bubble chart of top 25 metabolites ranked by VIP scores. **(G)** Box plots of representative differentially expressed metabolites.

To investigate the metabolic reprogramming of VAT in obesity, we conducted untargeted metabolomic profiling using liquid chromatography-tandem mass spectrometry (LC-MS/MS) in both positive and negative ionization modes. A total of 6303 metabolites were identified, including 2572 metabolites in positive mode and 3731 metabolites in negative mode, which were merged for downstream analysis. Metabolites annotation and classification were carried out based on the Human Metabolome Database, with the chemical taxonomy analysis revealing diverse classes of metabolites, including lipids and lipid-like molecules (41.36%), organic acids and derivatives (17.88%), organoheterocyclic compounds (10.71%) and etc (**Figure 3A**). We next identified 191 significantly altered metabolites through differential expression analysis (VIP > 1.0, P < 0.05), including 110 upregulated and 81 downregulated metabolites in the obese group compared to the controls (**Figure 3B**, **Supplementary Table S5**). PCA analysis based on metabolite intensities revealed a clear separation between obese and control groups, with PC1 and PC2 accounting for 22.1% and 12.8% of total variance, respectively (**Figure 3C**). Further supervised multivariate analyses using PLS-DA and OPLS-DA confirmed robust group discrimination. In PLS-DA, component 1 and component 2 explained 21.7% and 9.2% of the variance, respectively (**Figure 3D**). In OPLS-DA, component 1 and component 2 explained 24.8% and 10.4% (**Figure 3E**), further supporting metabolic divergence between the two groups.

Ranking by VIP scores highlighted the top 25 metabolites (**Figure 3F**), with Diniconazole, 4-Vinylcyclohexene, (R)-(+)-Ropivacaine, Octanamide and Ropivacaine emerging as the top 5 metabolites. Among the representative metabolites, we showed that the levels of 5 metabolites, including the above ones, were increased in the obese group, whereas Asparagine-betaxanthin was notably downregulated (**Figure 3G**). These findings reveal significant alterations in VAT metabolomic profiles associated with obesity, indicating potential metabolic biomarkers and dysregulated pathways involved in adipose tissue dysfunction.

### Functional pathways analysis of visceral adipose tissue metabolites associated with obesity

**Figure 4:**
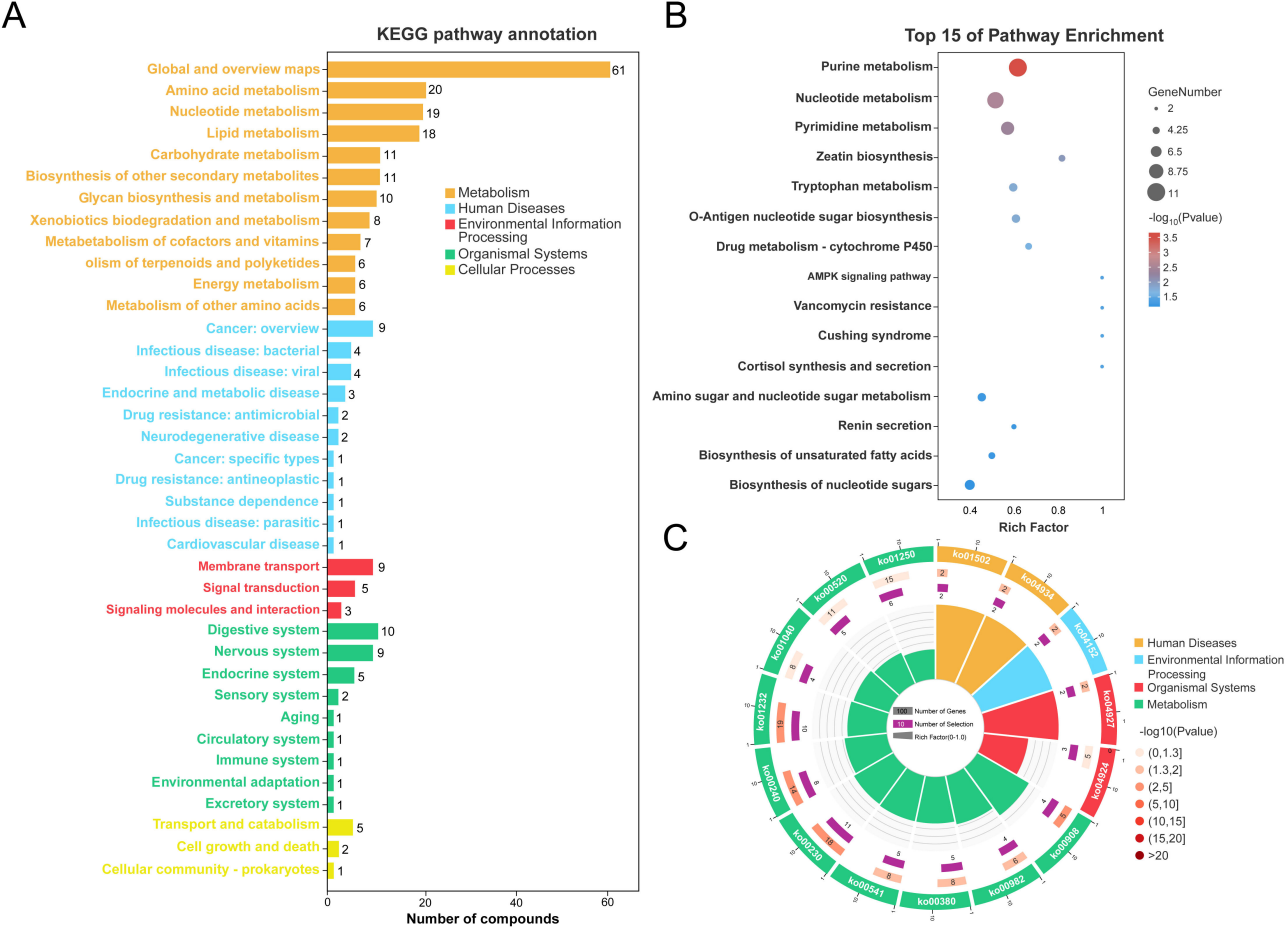
Functional annotation of adipose tissue metabolites in obesity vs. controls. **(A)** KEGG pathway classification of metabolites. **(B)** Bubble chart of top 15 enriched KEGG pathways. **(C)** Circular visualization of KEGG pathway enrichment (inner ring: numbers of KEGG terms, middle ring: −log_10_Pvalue, outer ring: KEGG pathway id).

To clarify the biological implications of the metabolomic changes observed in VAT from obese patients, we conducted functional annotation and pathway enrichment analyses using the KEGG database. Our classification of all identified differential metabolites via KEGG pathway analysis demonstrated their participation in a wide array of biological processes (**Figure 4A**). Specifically, the majority of metabolites were predominantly enriched in five major KEGG functional categories: Metabolism, Human Diseases, Environmental Information Processing, Organismal Systems, and Cellular Processes. Notably, the “Metabolism” category was predominant, with a particularly high representation in pathways related to amino acid metabolism (n = 20), nucleotide metabolism (n = 19), and lipid metabolism (n = 18) (**Supplementary Table S6**). These results underscore the profound metabolic reprogramming that occurs in obese VAT. The top 15 significantly enriched pathways, visualized by bubble plot (**Figure 4B**), further highlight key metabolic and disease-related processes. Purine metabolism, nucleotide metabolism, and pyrimidine metabolism were the most significantly enriched pathways, with the highest number of associated metabolites and the greatest statistical significance. Additional pathways of interest included tryptophan metabolism, AMPK signaling, and cortisol biosynthesis, suggesting potential disruptions in amino acid turnover, cellular energy sensing, and endocrine regulation in obesity. Circular visualization of pathway enrichment offers an integrated overview of pathway statistics, including the number of involved metabolites (inner ring), enrichment significance (−log_10_ P values; middle ring), and specific pathway IDs (outer ring) (**Figure 4C**). Consistent with the bubble plot, several metabolic pathways (e.g., ko00230: purine metabolism, ko00240: pyrimidine metabolism, ko00380: tryptophan metabolism) and human disease pathways (e.g., ko04924: cortisol synthesis and secretion) were highly enriched and statistically significant. The presence of signaling-related pathways (e.g., AMPK signaling) further supports a multifaceted metabolic dysfunction in obese VAT. Together, these analyses suggest that the visceral adipose tissue of obese individuals exhibits extensive alterations in core metabolic pathways, nucleotide turnover, and hormonal biosynthesis, which may underlie systemic metabolic disturbances associated with obesity.

### Association analysis between representative metabolites, proteins, and clinical phenotypes

**Figure 5:**
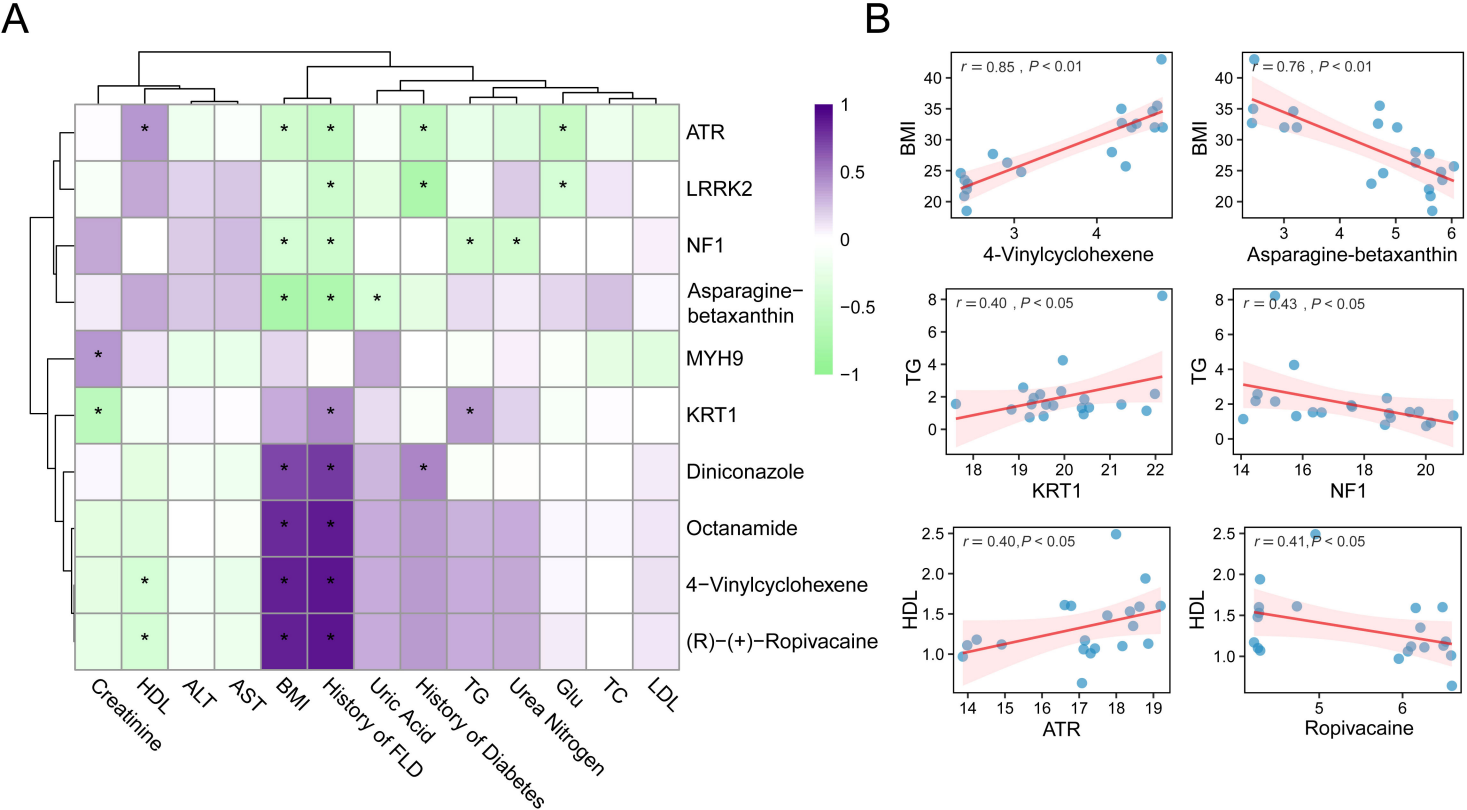
Correlation analysis between molecules and clinical phenotypes. **(A)** Heatmap of spearman correlations between proteins/metabolites and clinical parameters. **(B)** Scatter plots of representative correlations. * means P<0.05.

We performed an integrative correlation analysis between representative metabolites, proteins, and a panel of clinical phenotypes across VAT samples collected from bariatric surgery patients and healthy controls for investigating the biological relevance of the identified differential molecules. As shown in **Figure 5A**, a Pearson correlation heatmap was generated, where the columns represent selected proteins and metabolites with significant differential expression, and the rows correspond to key clinical indices, including BMI, blood lipid profiles (TG, HDL, LDL, TC), liver enzymes (ALT, AST), renal function markers (creatinine, urea nitrogen), glucose metabolism, and clinical histories (e.g., MASLD, diabetes) (**Supplementary Table S7**). The color scale reflects the direction and magnitude of correlation coefficients, while the asterisk indicates that the correlation coefficient is greater than 0.4. Notably, 4-Vinylcyclohexene showed a strong positive correlation with BMI (r = 0.85, P < 0.01), whereas asparagine-betaxanthin displayed a significant negative correlation with BMI (r = -0.76, P < 0.01). Other significant correlations included KRT1 with TG (r = 0.40, P < 0.05), NF1 with TG (r = -0.43, P < 0.05), ATR with HDL (r = 0.40, P < 0.05), and (R)-(+)-ropivacaine with HDL (r = -0.41, P < 0.05).

To enhance the visualization of these findings, scatter plots of six representative molecule-phenotype pairs with moderate to strong correlations are presented in **Figure 5B**. These plots reveal the direction and strength of the associations, underscoring the potential physiological roles of these biomolecules in metabolic regulation. Overall, the results demonstrate that specific metabolites and proteins identified in adipose tissue are closely linked to obesity-related clinical characteristics. These molecular correlations may indicate underlying changes in adipose tissue function and provide candidate targets for further mechanistic studies or therapeutic interventions.

### Integrated proteomic and metabolomic analysis reveals its core regulatory network

**Figure 6:**
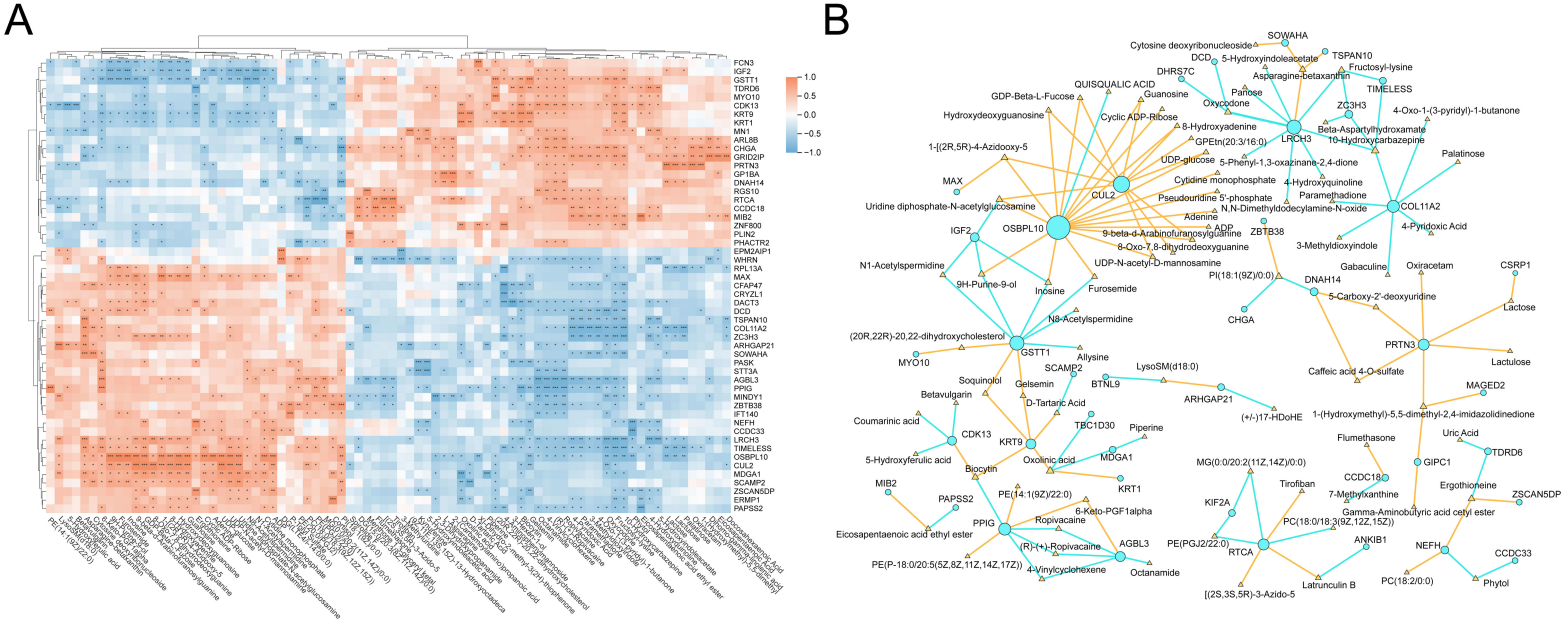
Integrative analysis of proteins and metabolites. **(A)** Heatmap of pearson correlations between DEPs and differential metabolites (|cor| > 0.7). **(B)** Interaction network of strongly correlated protein-metabolite pairs (nodes: proteins/metabolites, node color: yellow represents positive correlation, and blue represents negative correlation). * means P<0.05, ** means P<0.01, *** means P<0.001.

To explore the potential molecular interactions underlying metabolic alterations in visceral adipose tissue, we performed an integrative analysis of proteomic and metabolomic data based on Pearson correlation. Representative DEPs and metabolites were selected for pairwise correlation analysis across all samples. As shown in **Figure 6A**, we constructed a heatmap displaying all protein-metabolite pairs with correlation coefficients above 0.7, indicating strong associations. The heatmap highlights coordinated changes between specific proteins and metabolites, suggesting their potential co-regulation interplay in adipose tissue metabolism. Subsequently, we extracted these highly correlated pairs to construct a protein–metabolite interaction network, revealing a core regulatory module (**Figure 6B**). Within this network, several key proteins emerged as hub nodes, including OSBPL10 (Oxysterol binding protein like 10), CUL2 (Cullin 2), LRCH3 (Leucine rich repeats and calponin homology domain containing 3), and PRTN3 (Proteinase 3). These proteins were closely associated with multiple metabolites, which may imply that they play a central role in mediating the proteomic-metabolomic crosstalk in the VAT microenvironment. The identification of these protein hubs may reflect underlying pathways involved in dyslipidemia (OSBPL10), beige fat biogenesis (CUL2), fatty liver disease and insulin resistance (PRTN3) (31–33). This integrated network provides novel insights into the molecular architecture governing adipose tissue dysfunction in obesity and may guide further exploration of regulatory mechanisms and therapeutic targets.

## Discussions

In this study, we observed significant alterations in both the proteomic and metabolomic profiles of VAT in obese patients undergoing SG compared to individuals with normal body weight. These molecular signatures reflect a profound remodeling of adipose tissue biology. Previous studies have reported that weight loss can reduce adipocyte size and reverse obesity-associated inflammation and insulin resistance (34, 35). Consistent with these findings, our results revealed changes in proteins involved in lipid handling (e.g., Lipid droplet organization pathway) and energy sensing (e.g., Muscle system process pathway). Lipid droplets are key organelles for triglyceride storage in adipocytes, impaired degradation of PLIN2 in obesity can lead to abnormally enlarged lipid droplets, contributing to lipotoxicity (36, 37). In addition, muscle contraction could regulate energy expenditure and adipogenesis by AMPK signaling (38, 39). Additionally, the protein autophosphorylation pathway was found to be suppressed in post-surgical patients. Dysregulated protein phosphorylation profile underlies muscle insulin resistance in type 2 diabetes (40). Collectively, compared to controls, the VAT of obese patients following SG exhibits distinct molecular signatures in terms of lipid droplet dynamics, inflammatory status, energy metabolism, and insulin sensitivity.

Among the pathway alterations, the most striking were those related to lipid metabolism signaling. Functional annotation analysis of DEPs and metabolites revealed significant disturbances in pathways governing lipid droplet biogenesis and turnover. Enlarged lipid droplets, as a hallmark of adipocyte hypertrophy, are one of the key contributor to obesity-induced insulin resistance (41). In fact, evidences have shown that weight loss can reduce adipose tissue mass, alleviate lipid overload, and partially reverse type 2 diabetes (42, 43). Our findings suggest that SG may induce a coordinated metabolic shift away from lipid accumulation, characterized by reduced lipid droplet formation and enhanced lipid mobilization or oxidation. This is further supported by the observed upregulation of the muscle contraction signaling pathway, through which skeletal muscle activity activates the AMPK/PGC-1α axis, promoting mitochondrial biogenesis, increasing fatty acid oxidation, and mitigating obesity-associated metabolic dysfunction (44).

Inflammatory activity also underwent marked changes following obesity. Many of the DEPs identified in our study are involved in innate immune responses and protein-metabolite interaction networks centered on inflammatory mediators. Notably, the neutrophil-derived serine protease PRTN3 (proteinase 3) emerged as a key hub. PRTN3 promotes adipose inflammation by activating proinflammatory cytokines such as IL-1β (45). Importantly, experimental models have shown that PRTN3 is upregulated in the steatotic liver of obese mice, while PRTN3 knockout prevents hepatic steatosis and insulin resistance (46). In light of our findings, the differential level of PRTN3 suggests that weight-loss interventions may suppress neutrophil-driven inflammation in visceral adipose tissue. This suppression may help restore adipokine homeostasis and improve systemic insulin sensitivity. In addition, we observed that several differentially expressed metabolites were enriched in purine and pyrimidine metabolism pathways, which are known to reflect shifts in inflammatory signaling (47). Collectively, the reduction in proinflammatory proteins and metabolites likely reflects an attenuation of chronic adipose immune stress following weight loss.

Our findings also highlight key regulatory factors associated with adipocyte phenotypes. For example, OSBPL10 was identified as a critical node. OSBPL10 binds sterols and has been implicated in dyslipidemia (48). Its presence in our network may suggest that alterations in cholesterol sensing accompany VAT remodeling. Similarly, CUL2 emerged as another central hub. Recent studies have shown that silencing CUL2 in adipocytes stabilizes PRDM16, thereby strongly promoting fatty acid oxidation while simultaneously suppressing pro-inflammatory and pro-fibrotic pathways (49). The expression level of CUL2 in VAT following SG may reflect the mechanistic basis of weight-loss efficacy. Clinically, these molecular features may possess predictive or therapeutic relevance. For example, given that genetic variants in OSBPL10 affect serum lipid profiles, patients who fail to downregulate OSBPL10 postoperatively may be predisposed to persistent dyslipidemia or delayed hepatic lipid clearance. Likewise, the role of CUL2 in thermogenic regulation suggests that elevated CUL2 expression in VAT could indicate attenuated weight loss or reduced metabolic benefit. Equally important, the core gene PRTN3 is directly linked to the pathophysiology of MASLD. If patients with SG who show greater remission of MASLD also exhibit a larger reduction in adipose PRTN3 levels, it may indicate a mechanistic link between adipose immune signaling and hepatic outcomes. As a result, these proteins and their associated metabolites represent promising candidate biomarkers for tracking and potentially predicting postoperative trajectories, including BMI changes, insulin sensitivity, and MASLD progression.

Our integrative proteomic-metabolomic analysis reveals key drivers of adipose remodeling after bariatric surgery. Central proteins such as OSBPL10, CUL2, and PRTN3 link lipid metabolism, energy balance, and immune signaling. While these findings provide mechanistic insights and reveal potential therapeutic targets, several limitations warrant consideration. The absence of longitudinal VAT sampling due to ethical constraints necessitates cautious interpretation of metabolic improvements, requiring further validation through animal models and *in vitro* systems. Notably, the dynamic nature of VAT remodeling may be influenced by interindividual variability and postoperative lifestyle modifications, underscoring the need for: (1) extended clinical follow-up with serial tissue profiling; (2) functional validation of candidate biomarkers using organoid models; and (3) rigorous preclinical evaluation of identified targets. These investigations will be critical for translating our discoveries into clinical treatment targets and biomarkers for obesity.

## Conclusions

In summary, this study integrates proteomic and metabolomic analyses to elucidate the molecular remodeling of visceral adipose tissue (VAT) following sleeve gastrectomy (SG). Compared to normal-weight controls, obese patients undergoing SG exhibited significant alterations in protein and metabolite profiles within VAT. These changes predominantly involved coordinated regulation of key pathways, including lipid metabolism, inflammatory signaling, and insulin sensitivity. Notably, pathways related to lipid metabolism, lipid droplet organization, and protein autophosphorylation were markedly perturbed. Further analysis identified several hub proteins, such as OSBPL10, CUL2, and PRTN3, which may play pivotal roles in linking adipose tissue remodeling to postoperative clinical benefits, including improvements in MASLD. These hub proteins also hold potential as biomarkers for monitoring surgical outcomes. Collectively, the findings provide a theoretical framework and potential therapeutic targets for the development of non-surgical interventions that mimic the metabolic benefits of bariatric surgery. Future studies should explore how modulating these factors might replicate surgical benefits through less invasive strategies.

## Data Availability

The original contributions presented in the study are included in the article/**Supplementary Material**. Further inquiries can be directed to the corresponding authors.

## Supplementary material

Supplementary Table 1 Clinical phenotypic characteristics of obese patients and controls.

Supplementary Table 2 The results of differential protein analysis.

Supplementary Table 3 GO annotation analysis results of proteins.

Supplementary Table 4 KEGG annotation analysis results of proteins.

Supplementary Table 5 The results of differential metabolites analysis.

Supplementary Table 6 KEGG annotation analysis results of metabolites.

Supplementary Table 7 Correlation analysis results between key molecules and clinical information.
